# Spherical Aberration of Intraocular Lenses

**Published:** 2010-10

**Authors:** Majid Moshirfar

**Affiliations:** John A. Moran Eye Center, University of Utah School of Medicine, Salt Lake City, Utah, USA

Advancements in cataract surgery and intraocular lens (IOL) design have optimized the postoperative optical performance of the pseudophakic eye. One of the recent spotlights of IOL design has been formulating optical properties similar to a clear, young lens and addressing spherical aberration.

Spherical aberration in the human eye is a combination of the positive spherical aberration of the cornea,^1^–^3^ and the negative spherical aberration of the crystalline lens.^4^,^5^ In young eyes, the positive spherical aberration of the cornea is compensated by the negative spherical aberration of the lens; as a result, overall spherical aberration in the young eye is low.^2^,^3^,^6^ As the eye ages, the optical properties of the crystalline lens change,^4^,^7^ resulting in overall positive spherical aberration^2^,^8^,^9^ and decreased optical performance. Spherical aberrations generally reduce the contrast of the retinal image^10^,^11^ and affect visual performance, especially under mesopic conditions.^12^

Conventional spherical IOLs increase the positive spherical aberration in the eye following cataract extraction.^13^,^14^ In 2002, an aspheric IOL design was introduced to compensate for the positive spherical aberration of the cornea.^15^ Aspheric IOLs have been designed with an anterior prolate suface (Tecnis, Advanced Medical Optics), a posterior prolate surface (Acrysof IQ, Alcon Laboratories), or with both anterior and posterior prolate surfaces (Akreos AO, SofPort AO and L161 AO, Bausch & Lomb) and compensate for corneal spherical aberration to varying degrees.

In this issue of JOVR, a double-blind randomized controlled trial conducted by Jafarinasab et al^16^ compares spherical aberration and contrast sensitivity among 3 different types of aspheric IOLs (Tecnis, Akreos AO, and Acrysof IQ) and one spherical IOL (Sensar). Significantly higher spherical aberration was reported with the spherical IOL and the zero-aberration aspheric IOL as compered to the negative aberration aspheric IOLs, however this advantage was pupil-size dependent. With increased pupil size from 4 to 6 mm, an increase in spherical aberration was observed for all four types of IOLs, however significantly more with the spherical IOL. Contrast sensitivity function under mesopic conditions and at low spatial frequencies (1.5 to 3 cpd) was significantly higher in the Tecnis group as compared to the others. At higher spatial frequencies (12 to 18 cpd), Acrysof IQ worked significantly better. The authors concluded that the performance of aspheric IOLs is pupil dependent and that their function deteriorates to some extent under mesopic conditions, as there was no significant difference between spherical and aspheric IOLs in mesopic contrast sensitivity at 6 cpd.

Although this study is a well-designed clinical trial with interesting results, the readers should keep in mind that the best way to compare two groups with analysis of variance (ANOVA) is using post hoc tests such as Bonferroni adjustment of type one error. This is one of the reasons for discrepancies in the results among different studies. Another explanation could be different measurement protocols.

There are several studies comparing different types of spherical and aspheric IOLs under various conditions and with varying protocols. The readers should be careful about applying the results and accepting them as general rules.
